# Mitochondrial DNA release via mPTP and BAX/BAK drives inflammatory injury in intestinal ischemia reperfusion

**DOI:** 10.1186/s12964-025-02603-3

**Published:** 2025-12-24

**Authors:** Yixin Jing, Yiguo Zhang, Tulanisa Kadier, Ke Ding, Rong Chen, Qingtao Meng

**Affiliations:** Department of Anesthesiology, Renmin Hospital of Wuhan University, Wuhan, 430060 China

**Keywords:** IIR, mtDNA, MCU, mPTP, BAX/BAK

## Abstract

**Background:**

Intestinal ischemia reperfusion (IIR) is a challenging and life-threatening clinical condition, with disease progression closely linked to excessive inflammatory responses. As a potent activator of innate immunity, the mechanism underlying mitochondrial DNA (mtDNA) release across the mitochondrial membrane remains incompletely elucidated.

**Methods:**

In this study, an in vivo IIR model was established by clamping the superior mesenteric artery in male mice, and an in vitro hypoxia reoxygenation (HR) model was constructed using Caco-2 cells. Combining multiple techniques including RNA sequencing, subcellular organelle isolation, laser confocal imaging, siRNA transfection, protein cross-linking, Western blotting, enzyme-linked immunosorbent assay (ELISA), and quantitative real-time PCR (qPCR), the regulatory mechanisms of mtDNA release and its biological effects in IIR were systematically verified.

**Results:**

We found that IIR significantly induced an increase in cytosolic and circulating mtDNA levels, correlating with inflammatory cytokine production. Mechanistic studies revealed that calcium overload mediated by the mitochondrial calcium uniporter (MCU) triggered the opening of the mitochondrial permeability transition pore (mPTP). Meanwhile, the pro-apoptotic protein BAX was recruited to mitochondria and interacted with BAK to form outer mitochondrial membrane oligomeric pores. Notably, although mPTP opening was independent of the BAX/BAK pathway, the two pathways exhibited sequential synergistic effects during mtDNA release. Inhibition of either pathway significantly reduced mtDNA release, decreased inflammatory cytokine levels, and alleviated intestinal tissue injury caused by IIR.

**Conclusions:**

These findings identify mtDNA as a potential biomarker for IIR and highlight the MCU-mPTP-BAX/BAK axis as a therapeutic target.

**Graphical abstract:**

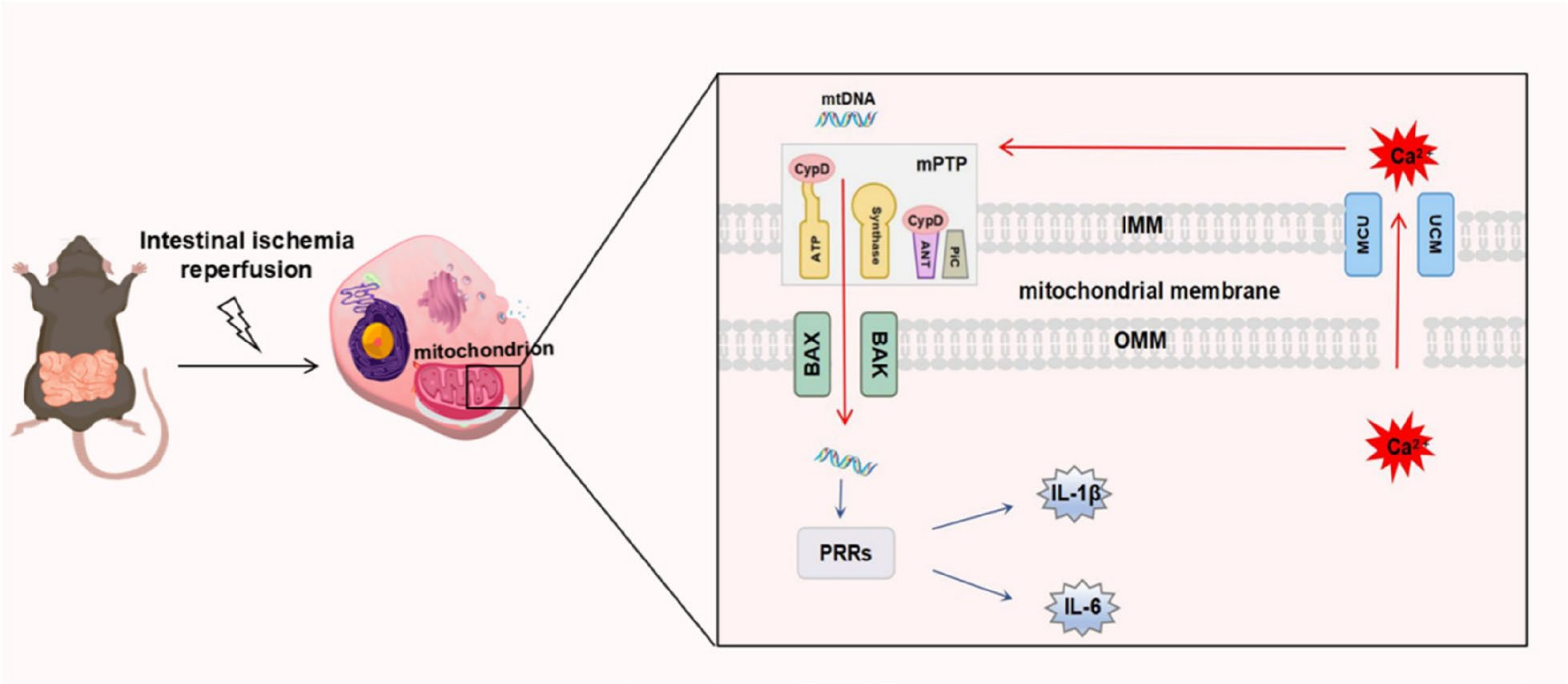

**Supplementary Information:**

The online version contains supplementary material available at 10.1186/s12964-025-02603-3.

## Background

Intestinal ischemia-reperfusion (IIR) is a life-threatening condition that often occurs secondary to various clinical scenarios, including acute mesenteric ischemia, shock, severe burns, and heart failure [1]. Despite advances in medical care, the in-hospital mortality rate of patients with intestinal ischemia has remained high over the past decades, with an increasing incidence reported [2]. Reduced intestinal perfusion leads to disruption of the intestinal barrier and microvascular dysfunction. Paradoxically, restoration of blood flow exacerbates tissue injury and triggers an uncontrolled inflammatory response, which may further progress to systemic inflammatory response syndrome (SIRS), multiple organ dysfunction syndrome (MODS), and even death [3]. However, there is limited understanding of the molecular mechanisms that lead to IIR injury, and there has been a lack of effective clinical methods for diagnosis and treatment.

Inflammation is an evolutionarily conserved process that protects the host from infection by eliminating pathogens and promoting tissue repair. However, if not properly terminated, inflammation transitions from a protective response to a driver of pathology [4]. A robust inflammatory cascade response is a key contributor to IIR progression, with a sterile inflammation role that cannot be ignored and is known to be mediated by mtDNA [5]. In mammalian cells, mtDNA is packaged into nucleoids within the mitochondrial matrix and encodes 13 essential subunits for oxidative phosphorylation. Due to their shared evolutionary ancestry with bacteria, mtDNA molecules retain hypomethylated CpG motifs that are sensed by classical pattern recognition receptors (PRRs) as “foreign” DNA, thereby activating innate immune pathways and promoting the production of pro-inflammatory cytokines such as IL-1, IL-6, and TNF-α [6]. These cytokines are widely accepted biomarkers of disease activity and mortality risk [7]. Studies have confirmed that patients suffering from severe trauma release massive quantities of mtDNA into the circulation, sometimes thousands of times higher than in healthy controls. Experimental models show that intravenous injection of mtDNA into mice induces hepatic and pulmonary injury along with systemic inflammation [8]. Similarly, intra-articular injection of mtDNA—but not nuclear DNA—elicits local cytokine production [9]. These findings suggest that mtDNA is not merely a biomarker but an active driver of inflammation, and targeting mtDNA-induced immune activation may offer a promising therapeutic strategy for IIR.

Although the release of mitochondrial DNA (mtDNA) is a key link between ischemia reperfusion and inflammation, the molecular mechanism by which mtDNA crosses the mitochondrial membrane under pathological conditions remains unknown. The mitochondrial permeability transition pore (mPTP) is a common terminal event in various pathological conditions, such as oxidative stress, and its opening provides a potential pathway for the escape of mtDNA into the cytoplasm. mPTP pore diameter is approximately 2–3 nm, allowing solute molecules with a molecular weight of less than 1.5 KDa to freely shuttle across the mitochondrial membrane [10]. However, the exact composition and opening mechanism of mPTP remain controversial: existing studies suggest that adenine nucleotide translocase (ANT) and F1F0-ATPase (ATP synthase) function as independent channels mediating different forms of mPTP opening; in addition, CypD can bind to F1F0-ATPase subunits to drive conformational changes, thereby regulating mPTP opening. As a key trigger for mPTP activation, calcium ions bind to CypD to induce mPTP opening and facilitate transmembrane substance exchange in mitochondria [11–13]. Sustained opening of mPTP will lead to the release of mtDNA fragments, which in turn mediates inflammatory damage to tissues [14]. It is worth noting that in addition to crossing the mPTP of the inner mitochondrial membrane, mtDNA also needs to break through the barrier of the outer mitochondrial membrane to reach the cytoplasm. Regarding the mechanism of mtDNA release mediated by the permeabilization of the outer mitochondrial membrane, early research mainly originated from the exploration of the mitochondrial apoptosis pathway-the BAX protein is activated and translocated to the outer mitochondrial membrane after being stimulated, interacts with the BAK protein to form an oligomer, ultimately leading to an increase in the permeability of the outer mitochondrial membrane, prompting the release of cytochrome c and initiating the apoptotic cascade reaction [15]. Although a number of studies have confirmed that mtDNA can simultaneously enter the cytoplasm during this process [16, 17], the complete molecular mechanism of mtDNA release in the context of IIR still urgently needs in-depth analysis.

Based on this, this study reveals that during the process of ischemia reperfusion, the mitochondrial calcium uniporter (MCU) drives the opening of mPTP by regulating mitochondrial calcium overload; subsequently, the BAX protein is recruited to the outer mitochondrial membrane and interacted with BAK to undergo oligomerization, ultimately promoting the release of mtDNA from the mitochondrial matrix into the cytoplasm, thereby inducing an inflammatory response.

## Methods

### IIR model and drug treatment

Healthy, wild-type, SPF-grade male C57BL/6J mice (aged 9-12 weeks, weighing 20–25 g) were purchased from Beijing Vital River Laboratory Animal Technology Co., Ltd. After one week of adaptive feeding, mice were fasted for 12 h before surgery (water was not restricted). Mice were anesthetized by intraperitoneal injection of pentobarbital sodium solution (50 mg/kg), and the abdomen was shaved and disinfected. A midline laparotomy was performed, and the root of the superior mesenteric artery (SMA) was clamped using a microvascular clip. After observing for 2 min to confirm complete occlusion of blood flow, the incision was sutured. After 45 min of ischemia, the abdomen was reopened at the original incision site, and the clip was released to allow reperfusion. Blood and intestinal tissues were collected 2 h after reperfusion for subsequent analyses.

In the Sham group, mice underwent laparotomy without vascular clamping, with other procedures identical to the IIR group. In the CsA pre-treatment group, mice were intraperitoneally injected with 5 mg/kg cyclosporine A (MCE, HY-B0579, USA) 30 min before surgery [18]. In the MSN pre-treatment group, mice were intraperitoneally injected with 5 mg/kg MSN-125 (MCE, HY-120079, USA). Control mice were injected intraperitoneally with DMSO.

###  mtDNA and nDNA Preparation and intraperitoneal injection in mice [19]

Mitochondrial and nuclear fractions were isolated from the livers of wild-type C57BL/6 mice using a tissue mitochondrial isolation kit (Beyotime, C3606, China). mtDNA and nDNA were then purified from mitochondrial pellets and nuclear fractions, respectively, with a genomic DNA extraction kit (Beyotime, D0063, China). Both DNAs were dissolved in sterile 0.9% sodium chloride solution (NS), and their concentrations and purity were determined using a NanoDrop. nDNA (5 mg/kg), mtDNA (5 mg/kg), or NS were administered via intraperitoneal (ip) injection in a 200µL volume, with samples collected 2 h later.

### Cellular hypoxia reoxygenation (HR) model and drug treatment

The human colon carcinoma cell line Caco-2 was purchased from Wuhan Procell Life Technology Co., Ltd. Cells were cultured in MEM medium supplemented with 20% fetal bovine serum and 1% penicillin-streptomycin in a humidified incubator at 37℃ with 5% CO_2_. To simulate hypoxia, cells were placed in a hypoxic chamber with 5% CO_2_, 1% O_2_, and 94% N_2_ for 12 h, followed by reoxygenation under normoxic conditions for 4 h. In the CsA pre-treatment group, 1 µM CsA was added 1 h before hypoxia modeling, after which the medium was replaced for subsequent operations.

### Ethidium bromide (EtBr) induced mtDNA depletion model

Caco-2 cells were cultured in medium supplemented with 1 mM sodium pyruvate (Servicebio, G4212, China) and 50 µg/mL uridine (Yuanye, S18067, China). EtBr (Thermo Fisher, 15585011, USA) was added to the medium at 1 µg/mL for 2 weeks to induce mtDNA depletion. mtDNA depletion was assessed by qPCR. Primer sequences are listed in Sect. 2.4.7.

### Detection indicators and methods

#### HE Staining, transmission electron microscopy (TEM), and immunohistochemistry (IHC) of intestinal tissue

Intestinal tissues were paraffin-embedded, sectioned, and stained with hematoxylin and eosin. Histopathological changes in the small intestine were observed and imaged under an Olympus microscope, and analyzed using CaseViewer 2.4 software. Chiu’s scoring system was used to assess mucosal injury as follows: 0 (Normal villi structure), 1 (Mild separation between villous tips and lamina propria), 2 (Moderate separation, with further widening of the gap, 3 (Extensive epithelial shedding from villous sides), 4 (Complete epithelial loss, lamina propria exposed), 5 (Structural disintegration of the lamina propria).

For TEM, fresh intestinal tissues were fixed with 2.5% glutaraldehyde and 1% osmium tetroxide, dehydrated using uranyl acetate, and then observed under a transmission electron microscope to obtain images of mitochondrial structures. Six animal samples were randomly selected from each experimental group, 3 different intestinal tissue regions were chosen from each sample for TEM sectioning, ensuring that the total number of mitochondria counted per animal sample was ≥ 300.The methods for mitochondrial morphological analysis are as follows:

i. Calculation method for Swollen Mitochondria Ratio (SMR): The percentage of swollen mitochondria in each transmission electron microscopy (TEM) field of view relative to the total number of mitochondria in that field. Criteria for identifying swollen mitochondria: Mitochondria were identified as swollen when their volume exceeded twice the average volume of normal mitochondria and their cristae structures appeared blurred.
ii. Cristae score [1]: Mitochondria were scored from 0 to 4 based on the abundance and morphology of mitochondrial cristae: Score 0 (no clearly distinguishable cristae); Score 1 (≥ 50% of the mitochondrial area without cristae coverage); Score 2 (≥ 25% of the mitochondrial area without cristae coverage); Score 3 (abundant but irregular cristae, covering > 75% of the mitochondrial area); Score 4 (abundant and regular cristae).
iii. Cristae density: Quantified by ImageJ software through measuring mitochondrial area and tracing cristae length. Cristae density = Total cristae length (µm) ÷ Mitochondrial cross-sectional area (µm²).
  For IHC, paraffin sections were incubated with antibodies against TFAM or Occludin to analyze protein levels in tissues.

#### RNA extraction and quantification

Total RNA was extracted using the RNA extraction kit (Beyotime, R0027, China). mRNA was reverse transcribed to cDNA using the ABScript III RT Master Mix for qPCR kit (ABclonal, RK20428, China). qPCR was performed on a Roche Lightcycler 4800II system with a 20µL reaction volume. Expression levels of *IL-1β* and *IL-6* were normalized to β-actin. All primers were synthesized by Sangon Biotech (Shanghai). Primer sequences are as follows:

**Table:** 

Gene	Sequences	
Mouse *IL-1β*	F	5’-TCGCAGCAGCACATCAACAAGAG-3’
	R	5’ -AGGTCCACGGGAAAGACACAGG-3’
Mouse *IL-6*	F	5’-TGGTGTTGCCTGCTGCCTTC-3’
	R	5’-GCTGAGATGCCGTCGAGGATG-3’
Mouse *β-actin*	F	5’-TATGCTCTCCCTCACGCCATCC-3’
	R	5’-GTCACGCACGATTTCCCTCTCAG-3’
Human IL-1β	F	5’-CCACCTCCAGGGACAGGATA-3’
	R	5’-TCAACACGCAGGACAGGTAC-3’
Human IL-6	F	5’-AGTGAGGAACAAGCCAGAGC-3’
	R	5’-GGTCAGGGGTGGTTATTGCA-3’
Human β-actin	F	5’-CCTGGCACCCAGCACAAT-3’
	R	5’-GGGCCGGACTCGTCATAC-3’

#### Circulating mtDNA detection

Serum was obtained after allowing blood to clot for 30 min at room temperature and centrifuging at 3000 rpm for 20 min. Total DNA was extracted using a genomic DNA extraction kit (Beyotime, D0063, China). Levels of mtDNA fragments (16S rRNA, ND1, COX2) were normalized to the nuclear gene HK2. Primer sequences were obtained from references [20, 21], and synthesized by Sangon Biotech (Shanghai) Co., Ltd. The primer sequences are as follows:

**Table:** 

Gene	Sequences	
Mouse 16S rRNA	F	5’-CCGCAAGGGAAAGATGAAAGAC-3’
	R	5’-TCGTTTGGTTTCGGGGTTTC-3’
Mouse ND1	F	5’-CTAGCAGAAACAAACCGGGC-3’
	R	5’-CCGGCTGCGTATTCTACGTT-3’
Mouse COX2	F	5’-ATAACCGAGTCGTTCTGCCAAT-3’
	R	5’-TTTCAGAGCATTGGCCATAGAA-3’
Mouse HK2	F	5’-GCCAGCCTCTCCTGATTTTAGTGT-3’
	R	5’-GGGAACACAAAAGACCTCTTCTGG-3’

#### Enzyme-linked immunosorbent assay (ELISA)

Serum levels of IL-1β and IL-6 were measured using ELISA kits (Thermo Fisher, 88–7013 A-88, 88–7064−88, USA) according to the manufacturer’s instructions. Absorbance at 450 nm was measured using a microplate reader.

#### Serum biochemical analysis

Serum samples were analyzed using an automatic biochemical analyzer (Olympus, Tokyo, Japan) for levels of alanine aminotransferase (ALT), aspartate aminotransferase (AST), creatinine (CREA), and urea (UREA).

#### Lactate dehydrogenase (LD) and malondialdehyde (MDA) content assay

Homogenates of intestinal tissues were prepared according to the kit instructions. LD content was measured using a colorimetric assay kit (Nanjing Jiancheng Bio, A019-2-1, China). MDA levels were measured using a lipid peroxidation assay kit (Beyotime, S0131S, China).

#### Subcellular fractionation and cytosolic mtDNA quantification

The method for cytoplasmic fractionation and mtDNA extraction in cells was performed as previously described [22]. Divide 2×10^5^ Caco-2 cells into two equal portions. Resuspend one of the portions in 200 µL of PBS, and extract the DNA using a genomic DNA extraction kit, which serves as the total mtDNA for the normalization control. Resuspend the second equal portion in 200 µL of a buffer containing 150 mM NaCl, 50 mM HEPES (pH 7.3), and 20 µg/mL digitonin (Beyotime, ST1272, China). After mixing the homogenate for 10 min, the procedure is terminated when more than 50% of the cells are positive as detected by trypan blue staining. Centrifuge the sample at 1000 g for 3 min for three times. Transfer the cytoplasmic supernatant to a new tube and centrifuge it at 17,000 g for 10 min. The supernatant is the cytoplasmic fraction, and the precipitate is the mitochondria.

Isolate the DNA from these cytoplasmic fractions using a genomic DNA extraction kit. The mtDNA from the whole-cell extract is used as the normalization control for the cytoplasmic mtDNA.The primer sequences of mtDNA were derived from Reference [23] and synthesized by Sangon Biotech (Shanghai) Co., Ltd.The primer sequences are as follows:

**Table:** 

Gene	Sequences	
Human ND1	F	5’-CACCCAAGAACAGGGTTTGT-3’
	R	5‘-TGGCCATGGGTATGTTGTTAA-3’
Human D-LOOP	F	5’-CTATCACCCTATTAACCACTCA-3’
	R	5’-TTCGCCTGTAATATTGAACGTA-3’
Human COX2	F	5’-AATCGAGTAGTACTCCCGATTG-3’
	R	5’-TTCTAGGACGATGGGCATGAAA-3’
Human ATP6	F	5’-AATCCAAGCCTACGTTTTCACA-3’
	R	5’-AGTATGAGGAGCGTTATGGAGT-3’

#### mPTP opening detection

mPTP opening was detected using a MPTP detection kit (Beyotime, C2009S, China), with concurrent mitochondrial-specific labeling via Mito-Tracker Red CMXRos (Beyotime, C1035, China). After cells were treated according to the experimental protocol, they were stained following the instructions. After staining, detection buffer was added, and cells were imaged via a laser scanning confocal microscope (Olympus FV1200). ImageJ software was used to analyze the average mPTP fluorescence intensity (total regional fluorescence intensity/area) and immunofluorescence colocalization.

#### Mitochondrial calcium detection

Rhod-2/AM (Thermo Fisher, R1244, USA) staining was performed according to the kit instructions. Cells were incubated with 2.5 µM Rhod-2/AM, Mito-Tracker GREEN (Beyotime, C1048, China) and Hoechst 33258 (Beyotime, C1011, China) at 37℃ for 30 min and observed under a laser scanning confocal microscope (Olympus FV1200). ImageJ software was used to analyze the fluorescence intensity and the status of immunofluorescence colocalization.

#### Lentiviral overexpression and siRNA transfection

Caco-2 cells were infected with TFAM gene overexpression lentivirus (Lentivirus (ZsGreen-Puro)/CMV-c3×FLAG-Tfam, titer 2.4 × 10⁸TU/mL) and overexpression control lentivirus (Lentivirus (ZsGreen-Puro)/CMV-NC, titer 2.5 × 10⁸TU/mL) (Nanjing Kreis Biotechnology Co., Ltd., China) according to the product instructions, so as to achieve the overexpression of TFAM gene. The overexpression efficiency was verified by detecting the mRNA expression level of TFAM using qPCR and its protein expression level by Western blotting.

Caco-2 cells were transfected with specific or control siRNA using Rfect V2 siRNA transfection reagent (BIOG, 11042, China). Knockdown efficiency was confirmed by qPCR and Western blotting. siRNA sequences targeting MCU, BAX, BAK, and negative controls were designed by BIOG (Changzhou, China).The siRNA sequences are as follows:

**Table:** 

Gene	Sequences	
Human MCU	F	5’-CGGCUUACCUGGUGGGAAUTT-3’
	R	5’-AUUCCCACCAGGUAAGCCGTT-3’
Human BAX	F	5’-CCAGCAAACUGGUGCUCAATT-3’
	R	5’-UUGAGCACCAGUUUGCUGGTT-3’
Human BAK	F	5’-CGACAUCAACCGACGCUAUTT-3’
	R	5’-AUAGCGUCGGUUGAUGUCGTT-3’

#### Protein crosslinking assay

To assess the aggregation of mitochondrial BAX/BAK dimers, cells were suspended in PBS containing 0.2 mM BMH (Thermo Fisher Scientific, USA; Product No. 22330) and incubated at room temperature for 45 min. The cross-linking reaction was terminated by adding 1 M DTT and incubating for 15 min. Subsequently, the samples were centrifuged at 12,000 rpm at 4 ℃ for 10 min, and mitochondrial isolation and protein extraction were performed. Non-reducing loading buffer (Servicebio, G2030-1ML, China) was added, and the mixture was denatured at room temperature for 30 min, followed by Western blotting analysis using an SDS-free electrophoresis system. For quantification, the “Area” value of each oligomeric peak was calculated using ImageJ software, and the sum of these values represented the total integrated density of the oligomeric bands.

VDAC1 crosslinking was performed similarly using 2 mM DSS (Thermo Scientific, A39267, USA) at room temperature for 30 min, and then subsequent procedures were performed.

#### Immunoprecipitation (Co-IP)

After lysing Caco-2 cells with NP40 (Abclonal Technology, RM00022, China) and protease inhibitors, the CypD antibody (Proteintech, 12716-1-AP, China) or ANT1 antibody (Proteintech, 15997-1-AP, China) was added to the lysate. After incubation overnight, a protein-antibody complex was formed. Then, protein A/G magnetic beads (ABclonal Technology, RM02915, China) were added to the sample, and the mixture was inverted and mixed for 8 h to enable the magnetic beads to bind to the antibody-protein complex. Subsequently, the magnetic beads were centrifuged, and the supernatant was carefully removed while the bead-protein complex was retained. Then, the complex was repeatedly washed to remove non-specific binding. Finally, the protein was separated from the magnetic beads using an elution buffer, and the purified protein was collected for further analysis.

#### Western blotting

The samples were lysed with RIPA buffer containing a protease inhibitor mixture. The lysates were centrifuged at 12,000 g for 20 min at 4℃. The protein concentration was quantified using a BCA Protein Assay Kit (Beyotime, P0009, China). The protein extracts were separated by SDS-PAGE, and the proteins were transferred onto a PVDF membrane. The membrane was incubated with the primary antibody overnight at 4 ℃. Then, the membrane was incubated with the corresponding secondary antibody. The primary antibodies used were as follows: anti-BAX (1:10000, Proteintech, 50599-2-Ig, China), anti-BAK (1:1000, Cell Signaling Technology, 12105, USA), anti-MCU (1:1000, Cell Signaling Technology, 14997 S, USA), anti-Occludin (1:1000, Cell Signaling Technology, 91131, USA), anti-TFAM (1:10000, Proteintech, 22586-1-AP, China), anti-COX IV (1:1000, Cell Signaling Technology, 4850, USA), anti-VDAC1 (1:1000, Beyotime, AF1027, China), anti-β-actin (1:1000, Cell Signaling Technology, 4967, USA). The protein abundance was measured using a Bio-Rad chemiluminescence instrument and an enhanced chemiluminescence (ECL) detection system. Protein quantification was performed by measuring the optical density using ImageJ software.

#### Immunofluorescence

Cells were cultured on coverslips, fixed with 4% paraformaldehyde, and then permeabilized with 0.1% Triton X-100. The fixed cells were incubated with a blocking solution containing 1% bovine serum albumin (BSA) and 5% normal goat serum for 1 h, and then incubated with primary antibodies overnight at 4℃. The cells were washed with PBS, incubated with secondary antibodies for 1 h at room temperature, and counterstained with DAPI. Images were acquired using a fluorescence microscope (BX53, OLYMPUS, Japan) or a laser - scanning confocal microscope (Eclipse Ti2, Nikon, Japan). The primary antibodies used were as follows: anti-DNA (1:200, sigma, AC-30-10, USA), anti-TOM20 (1:200, Cell Signaling Technology, 42406, USA), anti-MCU (1:2000, Cell Signaling Technology, 14997 S, USA).The fluorescent dyes used were as follows: Mito-Tracker Red CMXRos (Beyotime, C1035, China) was used according to the manufacturer’s instructions before paraformaldehyde fixation, and all subsequent operations were performed under light-proof conditions throughout.

#### Detection of CCK-8 viability in cells

Treated as per the experimental design, cells were analyzed using the CCK-8 kit (Beyotime, C0038, China) following the manufacturer’s instructions to determine OD values for assessing cell viability.

### Statistical analysis

All data were analyzed using GraphPad Prism 9 software. Quantitative results are presented as mean$\:\:\pm\:$ SD ($\:\stackrel{-}{\mathrm{x}}\pm\:\mathrm{s}$). Differences between groups were assessed using independent-sample t-test or one-way/two-way ANOVA, followed by Tukey’s post hoc test. A P value < 0.05 was considered statistically significant.

## Results

### IIR impairs the integrity of the intestinal epithelial barrier and triggers an inflammatory response

**Fig. 1:**
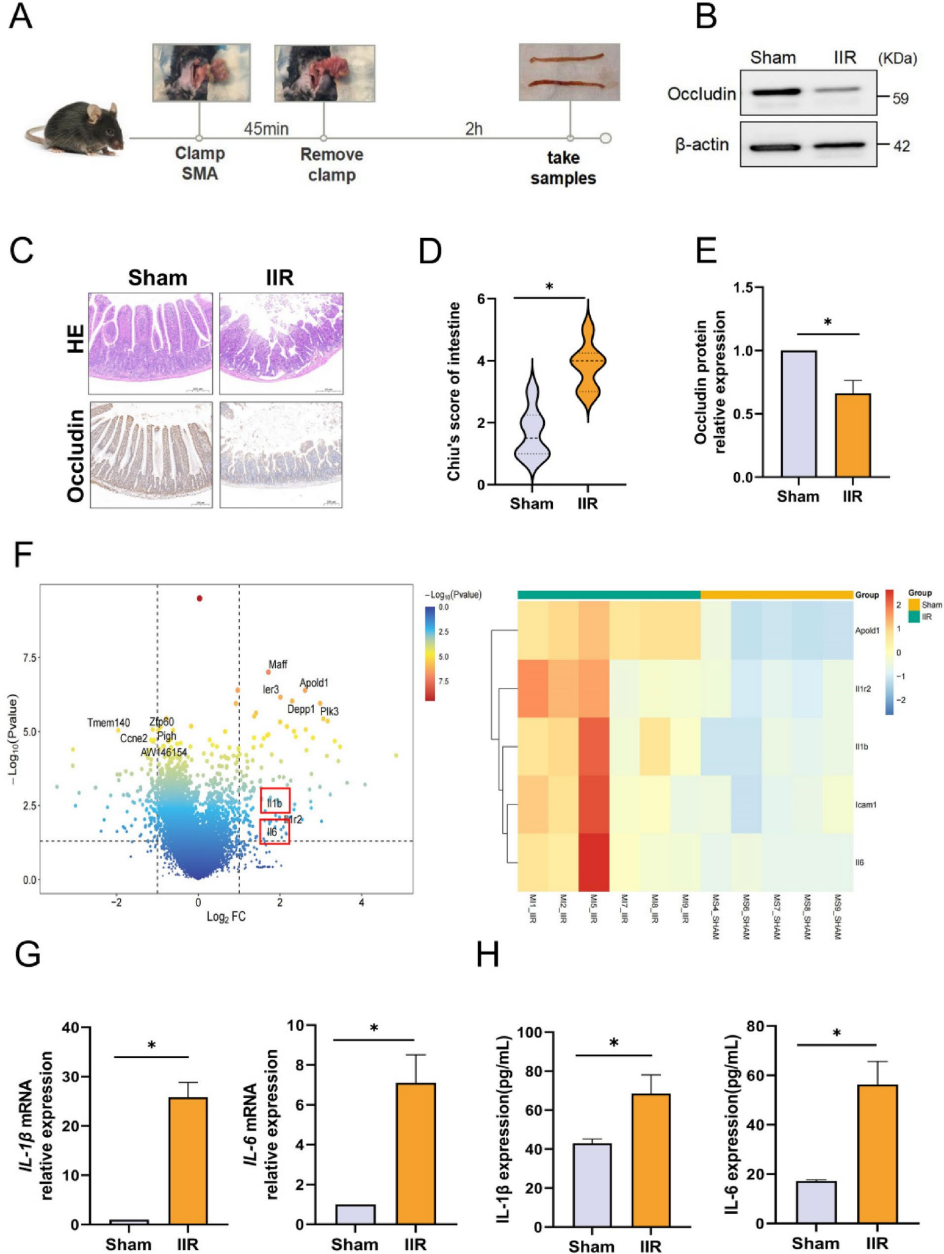
IIR impairs the integrity of the intestinal epithelial barrier and triggers an inflammatory response. **A** Schematic illustration of the mouse IIR model. **B**, **E** Western blotting analysis of Occludin protein expression levels in mouse intestinal tissues, *n* = 6. **C** Representative images of HE staining and Occludin immunohistochemical staining in mouse intestinal tissues, bar = 200 μm. **D** Chiu’s pathological score of murine intestinal tissues, *n* = 6. **F** RNA sequencing of intestinal tissues, heatmap and volcano plot for the analysis of differentially expressed genes related to tissue cytokines with a threshold of P-value < 0.05 and |log₂FC| > 1. **G** RT-qPCR analysis of *IL-1β* and *IL-6* mRNA levels in intestinal tissues, *n* = 6. **H** ELISA analysis of secreted IL-1β and IL-6 protein levels in murine plasma, *n* = 6. Data are presented as mean ± SD. *P < 0.05

We investigated the underlying mechanisms of IIR injury in a mouse model. After clamping the superior mesenteric artery, the color of the intestinal wall turned pale. Upon releasing the clamp after 45 min of ischemia and initiating 2 h reperfusion, significant intestinal wall edema was observed (Fig. 1A). To gain a better understanding of the structural and functional changes in the intestine following IIR, we examined intestinal tight junction protein Occludin. Western blotting analysis demonstrated that the expression of Occludin in the IIR group was significantly decreased compared with that in the Sham group (Fig. 1B and E), and this finding was further corroborated by immunohistochemical staining results (Fig. 1C). HE staining of intestinal tissues revealed that the intestinal mucosa in Sham group mice exhibited an intact structure, with regularly arranged, dense, and uniformly-sized villi. In contrast, in the IIR group, the intestinal mucosal structure was disrupted, characterized by villus sloughing and detachment, expansion of the subepithelial space, and an increase in lamina propria cells (Fig. 1C). The Chiu’s score in the IIR group was significantly higher than that in the Sham group (Fig. 1D).Collectively, these results indicate that IIR causes substantial damage to the intestinal barrier function and tissue structure in mice, suggesting the successful establishment of an IIR mouse model.

The inflammatory response has been identified as the core basis of ischemia reperfusion injury. To further explore the mechanisms of IIR injury, we performed RNA sequencing and transcriptomic analysis. IIR induced extensive alterations in gene expression (Fig.S1A), and the differentially expressed genes were significantly enriched in the innate immune signaling pathways (Fig.S1B). Multiple inflammation-related genes such as IL-1β and IL-6 were significantly upregulated in the IIR group (Fig. 1F). We detected the mRNA expression levels of IL-1β and IL-6 in intestinal tissues by RT-qPCR, and the results showed that the levels of IL-1β and IL-6 in the IIR group were significantly increased (Fig. 1G). The results of ELISA detection also confirmed that the protein contents of IL-1β and IL-6 in the circulation increased significantly after reperfusion (Fig. 1H). In conclusion, IIR significantly disrupts the structure of the intestinal epithelial barrier and activates a strong inflammatory response.

### mtDNA specifically exacerbates post IIR inflammatory injury

**Fig. 2:**
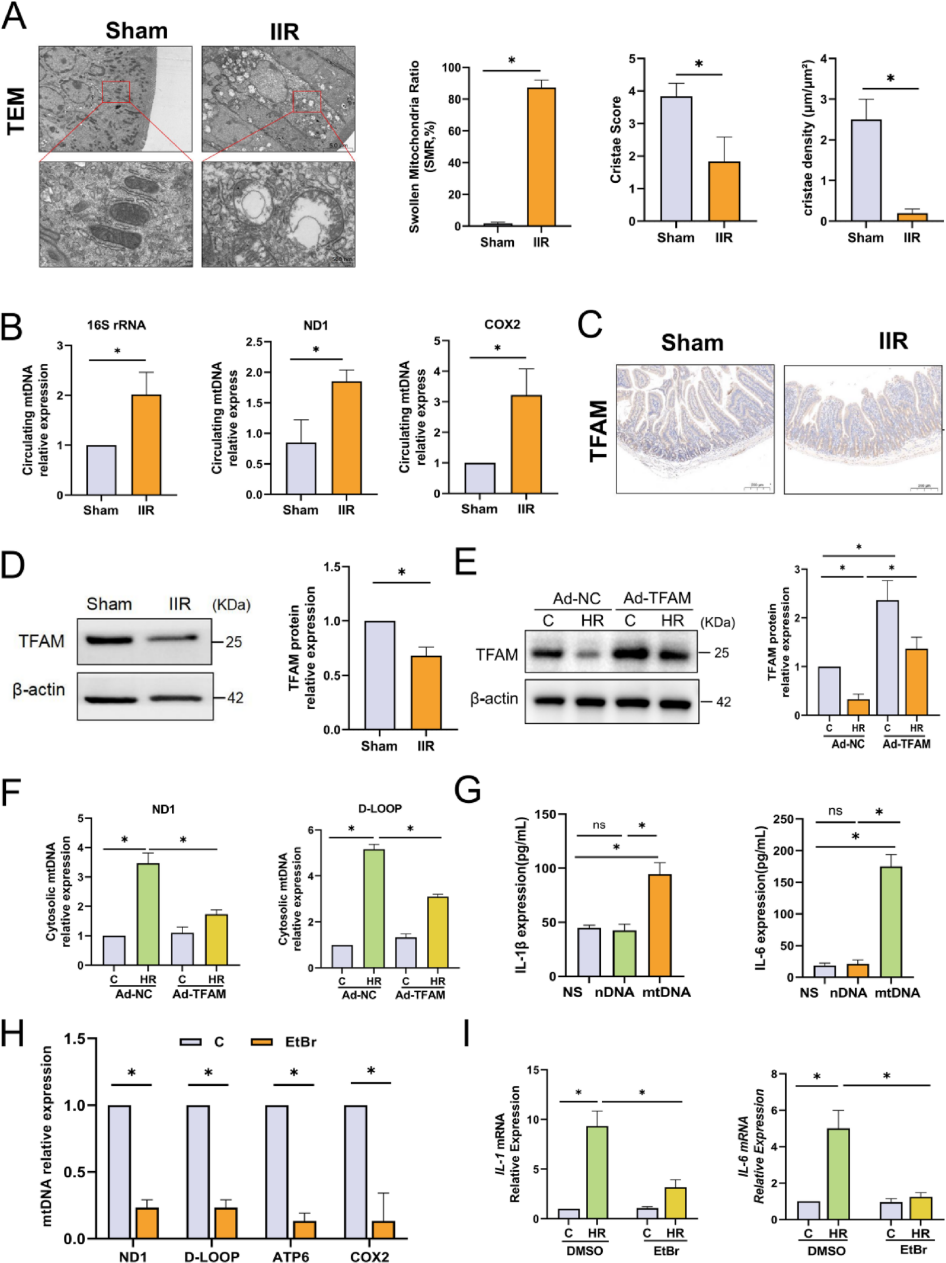
**A** Representative transmission electron microscopy (TEM) images of mouse intestinal tissues and quantitative analysis of Swollen Mitochondria Ratio (SMR, ratio of swollen mitochondria to total mitochondria), Cristae Score, and crista density, *n* = 6, scale bar = 5 μm/500 nm. **B** qPCR analysis of the expression levels of mtDNA fragments 16S rRNA, ND1, and COX2 in mouse plasma, *n* = 6. **C** Representative images of TFAM immunohistochemical staining in murine intestinal tissues, *n* = 6. **D** Western blotting analysis of TFAM protein expression levels in mouse intestinal tissues, *n* = 6. **E** Western blotting analysis of TFAM protein expression levels in Caco-2 cells transduced with Ad-NC or Ad-TFAM, *n* = 6. **F** qPCR analysis of cytoplasmic mtDNA fragment ND1, D-LOOP levels in Caco-2 cells, *n* = 6. **G** Mice were intraperitoneally (ip) injected with solutions containing nDNA (5 mg/kg), mtDNA (5 mg/kg), or normal saline (NS), and plasma samples were collected 2 h post-injection. Levels of secreted IL-1β and IL-6 proteins in mouse plasma were analyzed by ELISA, *n* = 6. **H** qPCR analysis of the expression levels of mtDNA fragments ND1, D-LOOP, ATP6, and COX2 in Caco-2 cells after EtBr depletion, *n* = 6. **I** RT-qPCR analysis of IL-1β and IL-6 mRNA levels in Caco-2 cells, *n* = 6. Data are presented as mean ± SD. *P < 0.05

The release of endogenous damage-associated molecular patterns (DAMPs) is a key factor in the inflammatory response induced by IIR injury. Existing studies have confirmed that mitochondria are the main source of DAMPs. After detection by transmission electron microscopy, we found that there were significant morphological and structural changes in mitochondria after IIR. The originally relatively uniform oval or elongated structure became swollen with a blurred outline. Extensive damage were observed in the outer membrane, and in some areas, the membrane structure was even absent. The mitochondrial cristae became sparse and disordered. We performed a morphological quantitative analysis of mitochondrial morphology. The results showed that after IIR, the Swollen Mitochondria Ratio in intestinal tissue was significantly increased, while the mitochondrial cristae score and cristae density decreased significantly(Fig. 2A). These changes indicate that the structure and function of mitochondria were severely damaged. mtDNA is the organellar genome of mitochondria, and it has been widely studied as a key initiator of sterile inflammation [24]. We detected the mtDNA content in the circulation of mice by qPCR and found that the level of mtDNA released into the blood after IIR increased significantly (Fig. 2B). Mitochondrial transcription factor A (TFAM) is a transcription factor that maintains the stability of mtDNA and initiates its replication. Multiple studies have shown that TFAM deficiency leads to abnormal packaging and cytoplasmic escape of mtDNA [22, 25]. We found through Western blotting analysis that the expression of TFAM in intestinal tissues decreased significantly after IIR (Fig. 2D), and this was also confirmed by immunohistochemistry (Fig. 2C). To clarify the pro-inflammatory role of mtDNA in IIR, mice were randomly assigned to three groups and received an intraperitoneal injection of normal saline (NS), nuclear DNA (nDNA), or mtDNA, respectively. Subsequent detection results revealed that, in comparison with the NS group, the mtDNA group exhibited a significant increase in the levels of pro-inflammatory cytokines IL-1β and IL-6 in the peripheral circulation. In contrast, the nDNA group showed no obvious elevation in the circulating levels of these two inflammatory factors. (Fig. 2G).

To further verify whether the production of inflammatory factors is related to the release of mtDNA, we used Caco-2 cells to simulate IIR through hypoxia reoxygenation (HR) in vitro. Cell viability detected by CCK-8 assay revealed that after HR, the viability of Caco-2 cells decreased significantly, by up to 50%. This confirms the applicability and reproducibility of the HR model in Caco-2 cells (Fig.S2A). We used lentivirus to overexpress TFAM at the cellular level, and the overexpression efficiency was verified by Western blotting (Fig. 2E). Subsequently, we detected the content of cytoplasmic mtDNA fragments, and the results confirmed that TFAM overexpression could inhibit HR-induced cytoplasmic mtDNA release(Fig. 2F). Besides, we depleted mtDNA in cells using ethidium bromide (EtBr). Low concentrations (0.1–2 µg/mL) of EtBr are compounds that can block the replication of mtDNA without affecting nuclear DNA [26]. After culturing with EtBr (1 µg/mL) for 2 weeks, the copy number of mtDNA was inhibited by approximately 70% (Fig. 2H). Subsequently, we detected the levels of cellular inflammatory factors by RT-qPCR and found that the mRNA levels of IL-1β and IL-6 in the EtBr + HR group were significantly lower than those in the HR group (Fig. 2I). The above results indicate that mtDNA may act as a key initiator of sterile inflammation and be involved in the inflammatory injury mediated by IIR.

### Calcium overload leads to mPTP opening and subsequently causes mtDNA release following HR

**Fig. 3:**
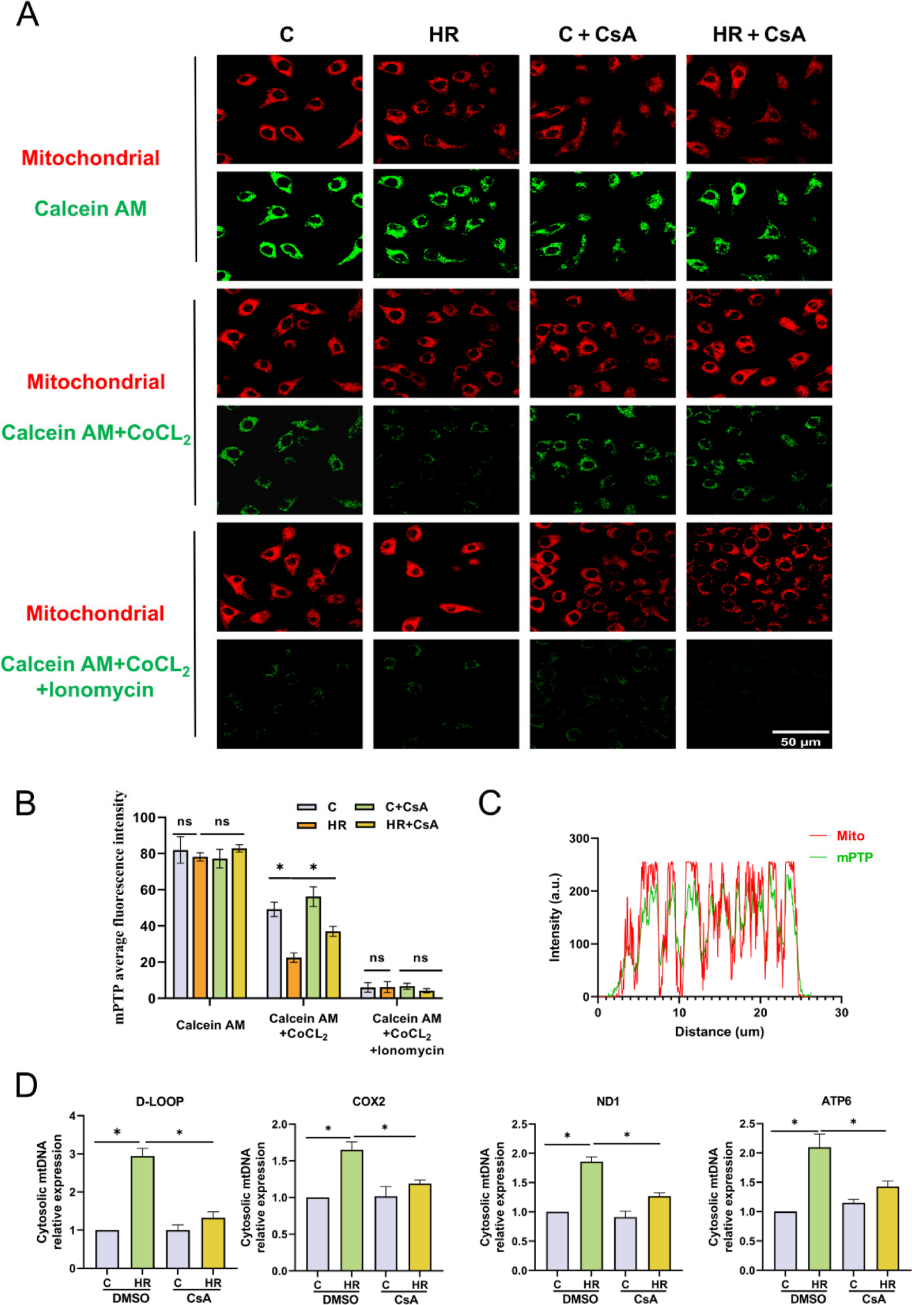
Calcium overload leads to mPTP opening and subsequently causes mtDNA release following HR. **A** Representative fluorescent images of mPTP detection in Caco-2 cells via Calcein AM assay. Mito-Tracker Red CMXRos (red) is the mitochondrial internal reference, and green fluorescence reflects mPTP opening level, *n* = 6; bar = 50 μm. **B** Quantitative analysis of average mPTP fluorescence intensity (total regional fluorescence intensity/regional area) in each group via Image J, *n* = 6. **C** Image J was used for quantitative analysis of immunofluorescence colocalization. **D** qPCR analysis of the expression levels of cytosolic mtDNA fragments ND1, D-LOOP, ATP6, and COX2 in Caco-2 cells after treatment with CsA, *n* = 6. Data are presented as mean ± SD. *P < 0.05

We detected the release of mtDNA into the circulation in mice, yet it remains unknown how mtDNA is induced to release into the cytosol. As the primary site for cellular biological oxidation and energy conversion, mitochondria play a crucial role during IIR. Among them, the continuous opening of the mitochondrial permeability transition pore (mPTP) under stress conditions will lead to the release of mtDNA and mediate tissue damage [27]. Caco-2 cells were incubated with the membrane-permeable fluorescent probe Calcein AM and the fluorescence quencher CoCl₂ to detect the opening degree of mPTP. The weaker the green fluorescence, the higher the opening degree of the pore. The results showed that compared with the C group, the average fluorescence intensity of mitochondria in the HR group was significantly decreased. Quantitative analysis indicated that HR could lead to a significant increase in the opening degree of mPTP. In addition, after increasing the intracellular calcium ion level by adding the calcium ionophore lonomycin after pre-incubation with Calcein AM and CoCl_2_, the average fluorescence intensity was significantly weakened, and there was no significant difference among the groups. This suggests that calcium ions are one of the important inducing factors for the opening of mPTP. To verify the role of mPTP in the release of mtDNA, we used CsA, a specific inhibitor of mPTP, inhibits the opening of the pore by specifically binding to CypD. The results showed that the pre-treatment with CsA could significantly inhibit the opening of mPTP (Fig. 3A, B). Based on Calcein AM + CoCl_2_ group, we performed colocalization analysis of mPTP fluorescence and mitochondrial red dye. The results indicated that mitochondria and mPTP showed significant colocalization in spatial distribution(Fig. 3C). We extracted the cytosolic components through subcellular organelle separation, and the qPCR detection results showed that the level of cytosolic mtDNA significantly increased after HR, while the inhibition of the mPTP pore could significantly suppress the release of mtDNA (Fig. 3D).The above results indicate that the opening of mPTP triggered by calcium overload during the HR process is the cause of the release of mtDNA.

### Mitochondrial calcium uniporter (MCU) mediated calcium overload regulates the opening of mPTP

**Fig. 4:**
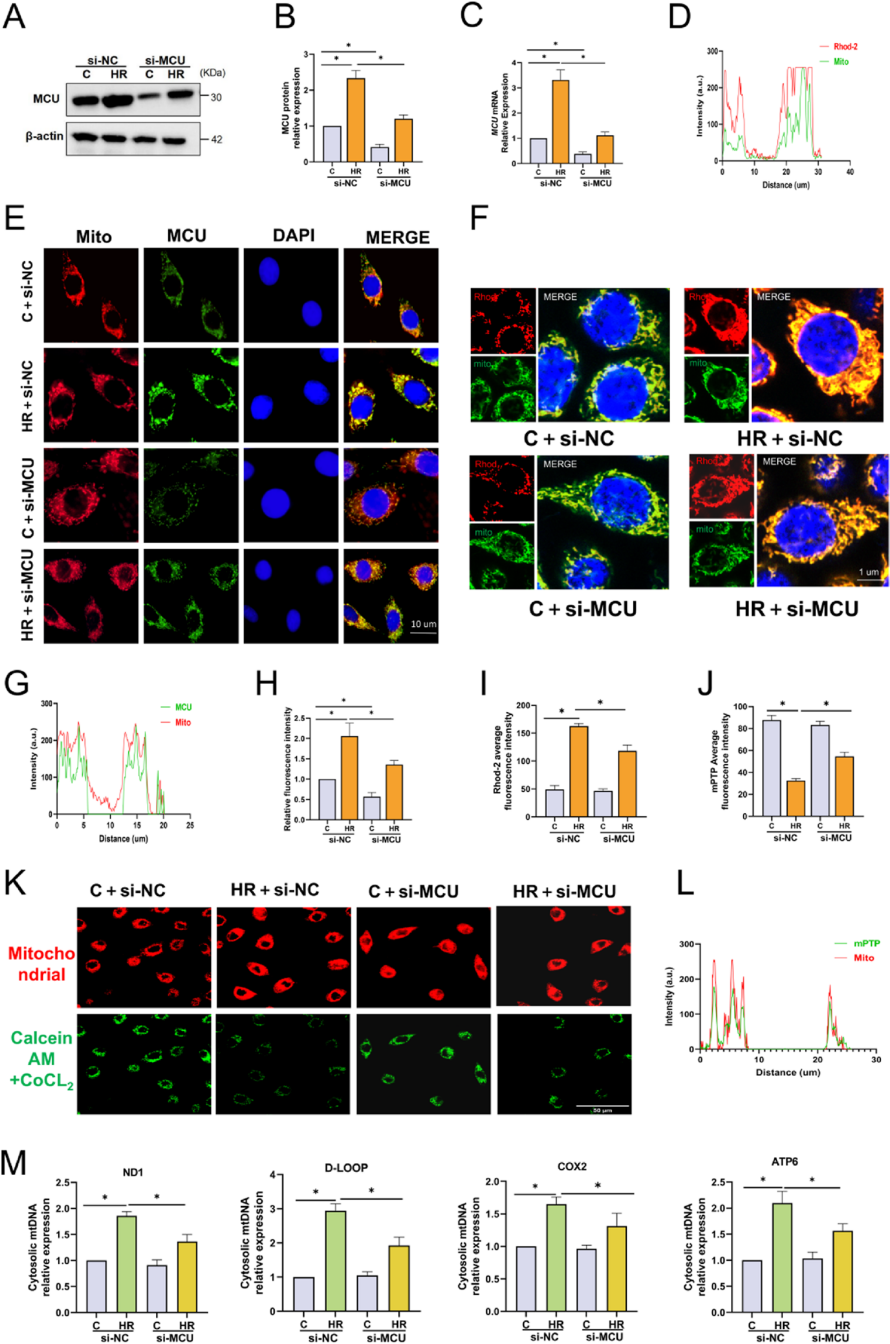
Mitochondrial calcium uniporter (MCU) mediated calcium overload regulates the opening of mPTP. **A**-**C** Western blotting (**A**,**B**) and RT-qPCR (**C**) analyses of MCU protein and mRNA expression levels in Caco-2 cells (si-MCU vs. si-NC), n = 6. **D** Representative immunofluorescence images of Rhod-2AM (calcium probe) and green mitochondrial fluorescent dye colocalization, and quantitative analysis using Image J, n = 6. **E**,**H** Representative immunofluorescence images of MCU (red), red mitochondrial fluorescent dye (green), and DAPI (nucleus, blue) (**E**), and quantitative analysis of MCU mitochondrial colocalization (**H**) in Caco-2 cells, n = 6, bar = 10 μm. **F**,**I** Representative immunofluorescence images of Rhod-2AM (red), green mitochondrial fluorescent dye (green), and Hoechst 33342 (blue) (**F**), and quantitative analysis of Rhod-2AM mitochondrial colocalization (**I**) in Caco-2 cells, n = 6, bar = 1 μm. **G** Image J-based quantitative analysis of immunofluorescence colocalization between MCU (red) and red mitochondrial fluorescent dye (green) in Caco-2 cells, n = 6. **J**,**K** Representative fluorescent images of mPTP detection (using calcein-AM/Co²⁺ probe) (**J**) and quantitative analysis of average fluorescence intensity (**K**) in Caco-2 cells, n = 6, bar = 50 μm. **L** Image J-based quantitative analysis of immunofluorescence colocalization between mPTP (labeled by calcein-AM, green) and red mitochondrial fluorescent dye (red) in Caco-2 cells, n = 6. **M** RT-qPCR analysis of cytosolic mtDNA fragment (ND1, D-LOOP, ATP6, COX2) expression levels in Caco-2 cells, n = 6.. Data are presented as mean ± SD. *P < 0.05

Mitochondrial calcium overload is considered to be the primary trigger for the opening of the mPTP during HR. MCU is a highly sensitive transporter for Ca²⁺ uptake, which is located on the inner mitochondrial membrane [27]. The results of Western blotting and RT-qPCR showed that the mRNA and protein expressions of MCU increased significantly after HR in cells(Fig. 4A-C), and the immunofluorescence staining for MCU also supported this finding (Fig. 4E, G, H). We detected the mitochondrial calcium content using Rhod-2/AM. The results showed that after HR, the fluorescence intensity of mitochondrial calcium ions increased significantly, indicating the occurrence of calcium overload (Fig. 4 D, F, I). To further explore the specific mechanism by which MCU regulates the opening of the mPTP pore, we downregulated the expression of MCU using specific siRNA (si-MCU) at the cellular level. After verification by Western blotting and RT-qPCR, the expression of MCU was significantly reduced following MCU knockdown (Fig. 4A-C). The knockdown of MCU significantly inhibited the mitochondrial calcium level (Fig. 4D, F, I), and simultaneously alleviated the opening degree of mPTP (Fig. 4 K, L, J) and the cytosolic release of mtDNA after HR (Fig. 4M). This suggests that the regulation of mitochondrial calcium overload by MCU may be the key to controlling the opening of mPTP.

### Oligomerization of BAX/BAK triggers the mitochondrial outer membrane permeabilization (MOMP) after HR

**Fig. 5:**
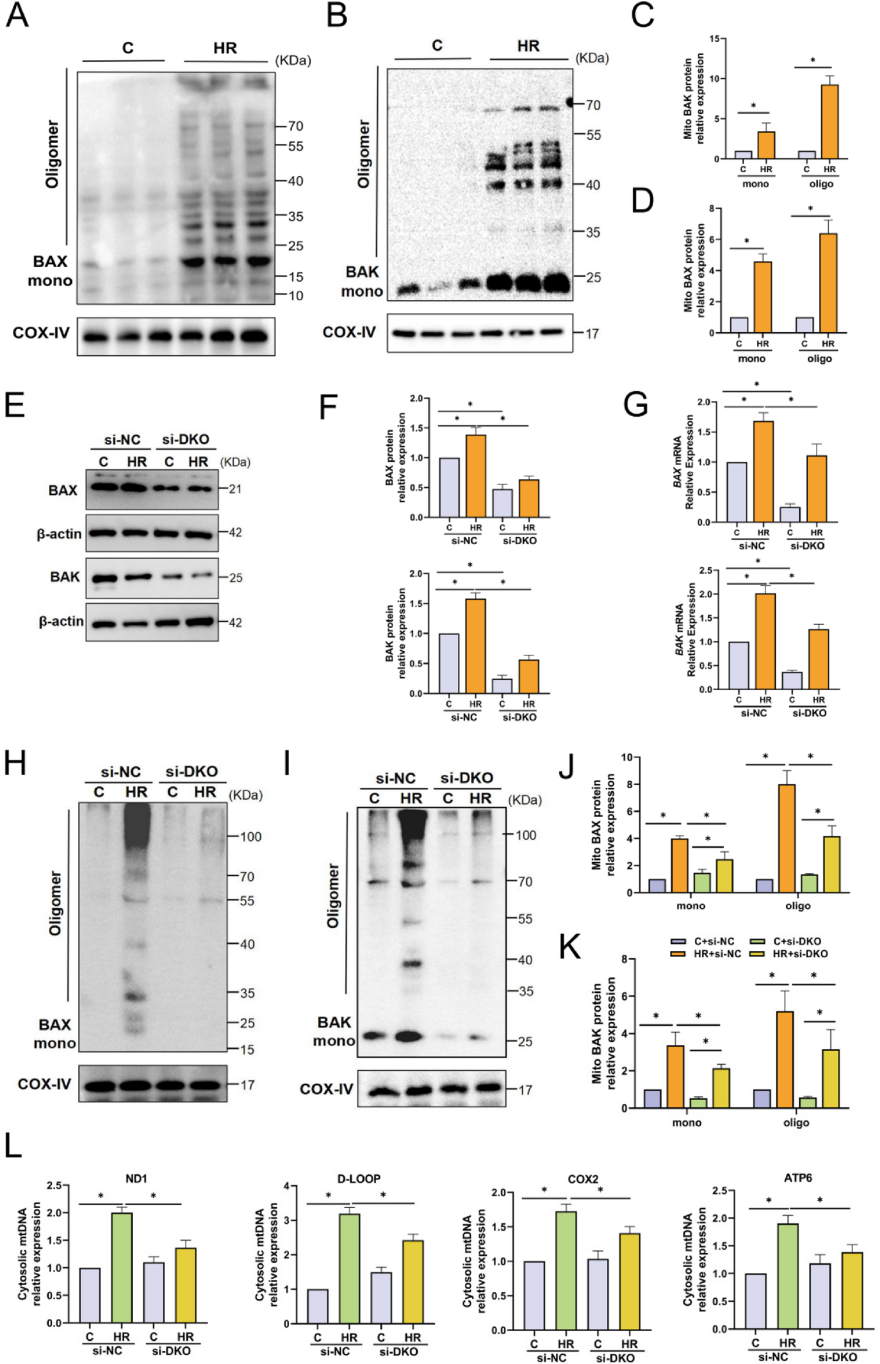
Oligomerization of BAX/BAK triggers the Mitochondrial Outer Membrane Permeabilization (MOMP) after HR. **A**-**D** Mitochondrial fractions were extracted through subcellular organelle separation. Western blotting analysis of the expression levels of monomeric and oligomeric forms of BAX and BAK proteins in mitochondria after BMH cross-linking treatment, n = 6. **E**-**G** Western blotting and RT-qPCR analysis of the protein and mRNA expression levels of BAX and BAK in Caco-2 cells, n = 6. **H**-**K** Mitochondrial fractions were extracted by subcellular separation. Western blotting analysis of the expression levels of monomeric and polymeric forms of BAX and BAK proteins in mitochondria after BMH cross-linking treatment, n = 6. **L** qPCR analysis of the expression levels of cytosolic mtDNA fragments ND1, D-LOOP, ATP6, and COX2 in Caco-2 cells, n = 6. Data are presented as mean ± SD. *P < 0.05

It has been reported that the mitochondrial recruitment and oligomerization of VDAC1 [28] and BAX [29] mediate the MOMP and are involved in the release of mtDNA. After using the amine-specific cross-linking agent DSS, we detected the expression of VDAC1 in the mitochondrial fraction. The Western blotting results showed that there was no difference in the expression of mitochondrial monomers of VDAC1 after HR, and no significant oligomers were formed (Fig.S3A,B). Under normal conditions, BAX is distributed in the cytoplasm, but it translocates to the mitochondria during stress and interacts with BAK to regulate and promote each other’s assembly. After using the thiol-specific cross-linking agent BMH, we detected the expression of BAX/BAK in the mitochondrial fraction. The Western blotting results showed that after HR, BAX was transferred from the cytoplasm to the mitochondria, oligomers of BAX and BAK appeared, and the expressions of both monomers and oligomers were significantly higher than those in the C group (Fig.5A-D). This indicates that the mitochondrial recruitment of BAX and the oligomerization of BAX/BAK may be involved in the MOMP during HR.

To determine whether the redistribution of mtDNA is dependent on BAX/BAK-mediated MOMP, we first used siRNA to knock down both BAX and BAK simultaneously (si-DKO). The knockdown efficiency of si-DKO was verified by Western blotting and RT-qPCR (Fig. 5E-G). After protein cross-linking treatment, we extracted mitochondria and performed Western blotting. The results showed that the si-DKO treatment significantly inhibited the mitochondrial recruitment of BAX and the oligomerization of BAX/BAK after HR (Fig. 5H-K). Similarly, the si-DKO treatment significantly alleviated the translocation of cytosolic mtDNA induced by HR (Fig.5 L). Overall, these data indicate that under HR conditions, matrix-localized mtDNA is released from mitochondria following BAX/BAK-dependent MOMP.

### Independent but sequential activation of mPTP and BAX/BAK leads to release of mtDNA following HR

**Fig. 6:**
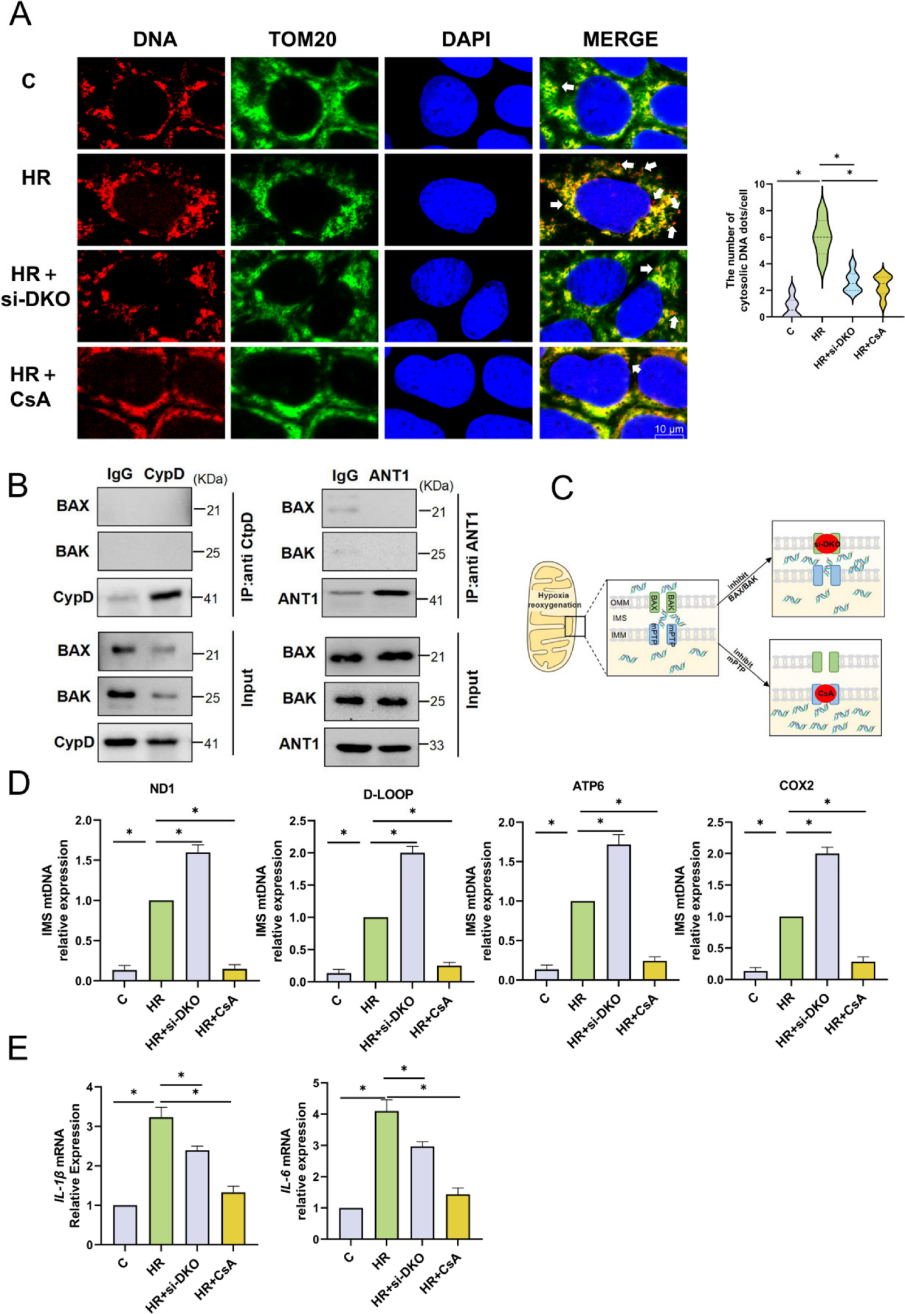
Independent but Sequential Activation of mPTP and BAX/BAK Leads to Release of mtDNA following HR. **A** Representative confocal immunofluorescence images and quantitative analysis of DNA, TOM20, and DAPI in Caco-2 cells, the number of cell= 10; bar = 10 μm. **B** Co-IP was performed using anti-CypD or anti-ANT1 antibodies, and BAX and BAK proteins were detected by Western blotting, n = 6. **C** Schematic diagram of the detection of mtDNA fragments in the IMS. **D** qPCR analysis of the expression levels of mtDNA fragments ND1, D-LOOP, ATP6, and COX2 in the IMS of Caco-2 cells, n = 3. **E** RT-qPCR analysis of the mRNA levels of IL-1β and IL-6 in Caco-2 cells, n = 6. Data are presented as mean ± SD. *P < 0.05

To visualize the roles of the mPTP and BAX/BAK in the cytosolic release of mtDNA, we performed immunofluorescent staining for DNA and mitochondria. Laser confocal scanning results showed that leaked mtDNA appeared in the cytoplasm after HR, and the amount of cytosolic mtDNA decreased after inhibiting the mPTP or BAX/BAK (Fig. 6A). To further explore the molecular mechanisms by which they function, we conducted Co-IP experiments targeting BAX/BAK and the key components of the mPTP (CypD and ANT1). However, our results indicated that there was no direct interaction between the mPTP and BAX/BAK (Fig. 6B). To dissect the role of mitochondrial membrane pores in mtDNA release, Xian et al. [28]exploited the difference in proteinase K sensitivity. They incubated purified mitochondria with 20 µg/mL proteinase K for 30 min to disrupt the outer mitochondrial membrane while preserving the inner mitochondrial membrane, thereby extracting the intermembrane space (IMS) components. We used this method (Fig. 6C) to measure the relative amount of mtDNA in the IMS after inhibiting the mPTP and BAX/BAK respectively. After blocking the opening of the mPTP with CsA, the number of mtDNA fragments in the IMS decreased significantly, but increased after inhibiting the oligomerization of BAX/BAK (Fig. 6D). This suggests that mtDNA first leaves the inner mitochondrial membrane (IMM) through the mPTP-dependent pore and enters the IMS, and then is released into the cytoplasm through the BAX/BAK oligomeric pore.

The mtDNA released into the cytoplasm can trigger the innate immune response as an endogenous damage - associated molecular pattern, mediating the production of inflammatory factors. We detected the mRNA levels of IL-1β and IL-6 in cells after HR by RT-qPCR. The results showed that the mRNA levels of IL-1β and IL-6 increased after HR compared with the C group, and were significantly reduced after inhibiting the mPTP and BAX/BAK (Fig. 6E).

### Inhibition of mtDNA release ameliorates IIR injury

**Fig. 7:**
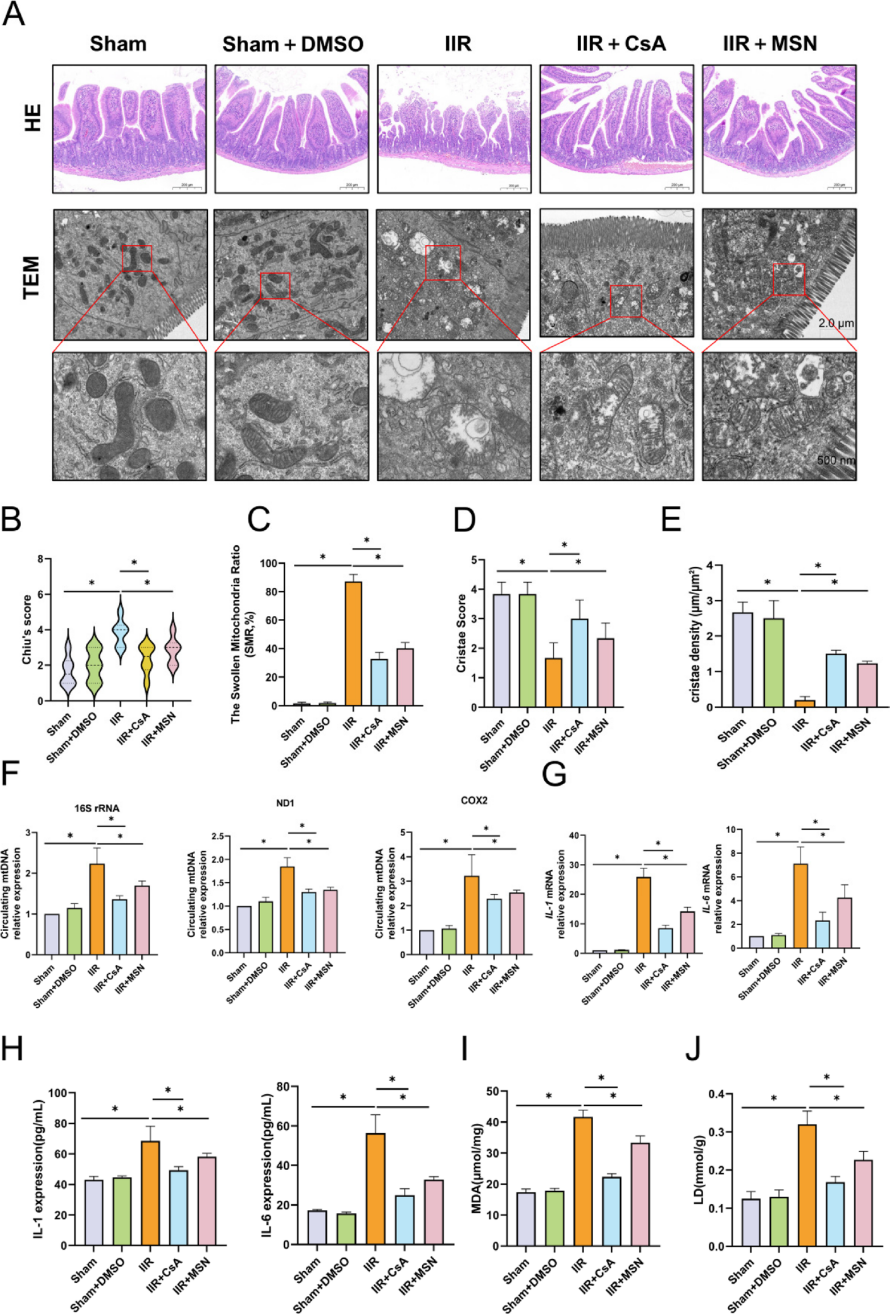
Inhibition of mtDNA release can ameliorate IIR injury. **A** Representative HE staining and electron microscopy images of murine intestinal tissues, bar = 200 μm, 2 μm/500 nm. **B** Chiu’s pathological scores of murine intestinal tissues, n = 6. **C**-**E** Quantitative analysis of Swollen Mitochondria Ratio (SMR, swollen mitochondria relative to the total number of mitochondria) and Cristae Score in murine intestinal tissues, n = 6. **F** qPCR analysis of the expression levels of mtDNA fragments 16S rRNA, ND1, and COX2 in murine plasma, n = 6. **G** RT-qPCR analysis of the mRNA levels of IL-1β and IL-6 in intestinal tissues, n = 6. **H** ELISA analysis of the secreted protein levels of IL-1β and IL-6 in murine plasma, n = 6. **I**-**J** Detection of the content of LDH and the activity of MDA in murine intestinal tissues, n = 6. Data are presented as mean ± SD. *P < 0.05

We have confirmed through in vitro experiments that mPTP and BAX/BAK oligomeric pores mediate the release of mtDNA. Next, we investigated the protective effects of the mPTP inhibitor CsA and the BAX/BAK oligomerization inhibitor MSN-125 in a mouse model. Intraperitoneal injection of CsA and MSN-125 had no significant effect on the liver and kidney functions (ALT, AST, CREA, UREA) of mice (Fig.S4A), which confirmed their safety for in vivo application. Subsequently, we examined the morphological changes of intestinal tissues and mitochondria in mice by HE staining and electron microscopy (Fig. 7A). The HE staining results showed that the use of CsA and MSN-125 alleviated the destruction of intestinal tissue structure caused by IIR and reduced the Chiu’s pathological score of intestinal tissues (Fig. 7B). The electron microscopy results of intestinal tissues also confirmed that CsA and MSN-125 mitigated mitochondrial structural damage after IIR, as evidenced by reduced mitochondrial swelling and preserved cristae integrity (Fig. 7C-E). Then, we detected the mtDNA content in the circulation of mice by qPCR. The use of CsA and MSN-125 reduced the release of mtDNA fragments in the circulation after IIR(Fig. 7F), and the levels of inflammatory factors in intestinal tissues and the circulation also decreased accordingly(Fig. 7G, H). In addition, the application of CsA and MSN-125 decreased the levels of LDH and MDA (Fig. 7I, J). LDH is a marker of general cellular injury, while MDA reflects lipid peroxidation; the reduction in both indices indicates that these treatments alleviated IIR-induced tissue damage to a certain extent.

## Discussion

IIR injury refers to the paradoxical exacerbation of tissue damage following the restoration of blood flow after a period of ischemia. Among its complex mechanisms, mtDNA-mediated inflammatory injury plays a pivotal role in disease progression and prognosis. Here, we demonstrated that mtDNA is released through the mPTP triggered by calcium overload mediated by the MCU, and then passes through the BAX/BAK oligomerization pores on the outer mitochondrial membrane and is released into the cytoplasm and circulation, ultimately driving inflammatory cytokine production in IIR.

Emerging evidence indicates that mtDNA leakage from stressed mitochondria serves as a potent pro-inflammatory signal, contributing to the progression of various inflammation-related pathologies [30]. In our study, we observed elevated levels of mtDNA in the cytosol and circulation following IIR. Depletion of mtDNA significantly reduced the secretion of key inflammatory cytokines, such as IL-1β and IL-6, whereas supplementation with exogenous mtDNA exacerbated cytokine production, underscoring a direct link between mtDNA and IIR-associated inflammation. These findings position mtDNA as a critical inflammatory driver and a potential biomarker for IIR.

The mPTP is a non-specific channel within the mitochondrial membrane that under physiological conditions permits rapid solute exchange between the mitochondrial matrix and cytosol. However, under stress, its persistent opening leads to the release of mitochondrial contents. Calcium overload is a well-established inducer of pathological mPTP opening [31], which has been observed in multiple models of organ ischemia reperfusion injury [32]. To interrogate the role of mPTP in IIR, we established a HR model using Caco-2 cells. In HR, mPTP opening was significantly elevated compared to controls and was further augmented by calcium ionophores, suggesting that calcium-mediated aberrant mPTP activation contributes to IIR pathogenesis. MCU serves as a critical regulator of mitochondrial and cellular calcium homeostasis. We found that HR exposure upregulated MCU expression, enhanced mitochondrial Ca²⁺ uptake, and activated mPTP opening, whereas MCU inhibition attenuated mitochondrial calcium overload and mPTP activation. These findings identify MCU-mediated mitochondrial calcium overload as a principal upstream event in mPTP opening. To explore the connection between mPTP activation and mtDNA release during IIR, we quantified cytosolic mtDNA levels and observed a significant increase following HR, which was mitigated by mPTP inhibition. Our results, consistent with prior reports, indicate that MCU-mediated calcium overload promotes mPTP-dependent mtDNA release during IIR.

Extensive studies have highlighted the importance of MOMP in mtDNA cytosolic release. We found that BAX/BAK, but not VDAC1, mediated MOMP in IIR. Under physiological conditions, BAX and BAK exist predominantly as inactive monomers. However, during apoptotic signaling, BAX/BAK oligomerize and integrate into the outer mitochondrial membrane [33]. Importantly, BAX/BAK pores not only initiate apoptosis but also facilitate mtDNA release to engage innate immune signaling. Recent studies have shown that BAX/BAK pores allow mtDNA to translocate into the cytosol [16, 29]. With BAX and BAK regulating each other’s assembly [17, 34]. Consistent with these findings, we observed mitochondrial recruitment and oligomerization of BAX with BAK following IIR. Knockdown of BAX/BAK effectively reduced cytosolic mtDNA levels and suppressed inflammatory cytokine production, further supporting the role of BAX/BAK-mediated membrane permeabilization in mtDNA release. Interestingly, this mechanism challenges the traditional view that mitochondrial apoptosis represents a non-inflammatory form of cell death. Although apoptosis has conventionally been regarded as immunologically silent compared to necrosis, accumulating evidence reveals new perspectives. For instance, apoptotic cells release ATP to act as a “find-me” signal for phagocytes, and high extracellular nucleotide concentrations can provoke pro-inflammatory responses [35]. Another possibility is that sublethal MOMP occurs under stress, leading to limited caspase activation insufficient to trigger cell death but sufficient to cause DNA damage and genomic instability [36]. Recent work further supports this paradigm, demonstrating that a minority of mitochondrial permeabilization during aging promotes mtDNA release via BAX/BAK pores, thereby initiating sterile inflammation. Together, these findings highlight BAX/BAK oligomerization-mediated mtDNA release as a potential therapeutic target in IIR, warranting future investigations.

Nevertheless, it is important to acknowledge several limitations. First, owing to technical constraints, we did not directly isolate mitochondria from mouse intestinal tissues under IIR conditions to assess cytosolic mtDNA release. Consequently, we inferred mtDNA release indirectly through decreased TFAM expression in intestinal tissues and elevated circulating mtDNA levels. This indirect assessment may not fully capture the dynamic changes of cytoplasmic mtDNA. Moreover, while the Caco-2 cell HR model is well-established in mechanistic research [1], it has limitations relative to intestinal organoid or primary intestinal epithelial cell models, which better recapitulate in vivo intestinal cellular heterogeneity and physiological specificity.

Second, although we preliminarily confirmed no direct interaction between BAX/BAK and mPTP key components (ANT1, CypD), a tight synergistic effect between BAX/BAK and mPTP was observed during IIR-mediated mtDNA transmembrane transport. This synergy is consistent with findings from Karch et al. [37] and Patel et al. [38], who demonstrated that BAX/BAK do not directly contribute to mPTP inner membrane channel composition or functional regulation, but act as functional components of the outer mitochondrial membrane components to modulate its activity. Additionally, mechanistic investigation of the ATP synthase c-subunit—another core mPTP subunit—was not included herein due to preliminary experimental constraints, including insufficient validation of specific antibodies against the ATP synthase c-subunit and limited applicability of functional tools. Further studies are needed to elucidate the upstream regulators coordinating this BAX/BAK-mPTP interplay.

Finally, while mtDNA is established as a key inflammatory activator in IIR, the exact interaction between mtDNA and pattern recognition receptors remains incompletely defined in our experiments. Future comprehensive in vivo studies and clinical validation will be critical to fully delineate these mechanisms and facilitate the identification of IIR therapeutic targets.

## Conclusions

Our present study concluded that mtDNA as a potential biomarker for IIR and highlight the MCU-mPTP-BAX/BAK axis as a therapeutic target.

## Supplementary Information

Supplementary Material 1

Supplementary Material 2

## Data Availability

No datasets were generated or analysed during the current study.

## Abbreviations

ALT: Alanine aminotransferase
AST: Aspartate aminotransferase
CREA: Creatinine
CypD: Cyclophilin D
DAMPs: Damage-associated molecularpatterns
EtBr: Ethidium bromide
ELISA: Enzyme-linked immunosorbent assay
HR: Hypoxia reoxygenation
IIR: Intestinal ischemia reperfusion
LD: Lactate dehydrogenase
MCU: Mitochondrial calcium uniporter
MDA: Malondialdehyde
mtDNA: Mitochondrial DNA
MOMP: Mitochondrial outer membrane permeabilization
mPTP: Mitochondrial permeability transition pore
MODS: Multiple organ dysfunction syndrome
PRRs: Pattern recognition receptors
qPCR: Quantitative real-time PCR
SIRS: Systemic inflammatory response syndrome
TFAM: Transcription factor A
