# Supplementary figures and images for: Mitochondrial DNA release via mPTP and BAX/BAK drives inflammatory injury in intestinal ischemia reperfusion
Source: Cell Commun Signal. 2025 Dec 24;24:50. doi: 10.1186/s12964-025-02603-3 (PMC12849455; doi:10.1186/s12964-025-02603-3)

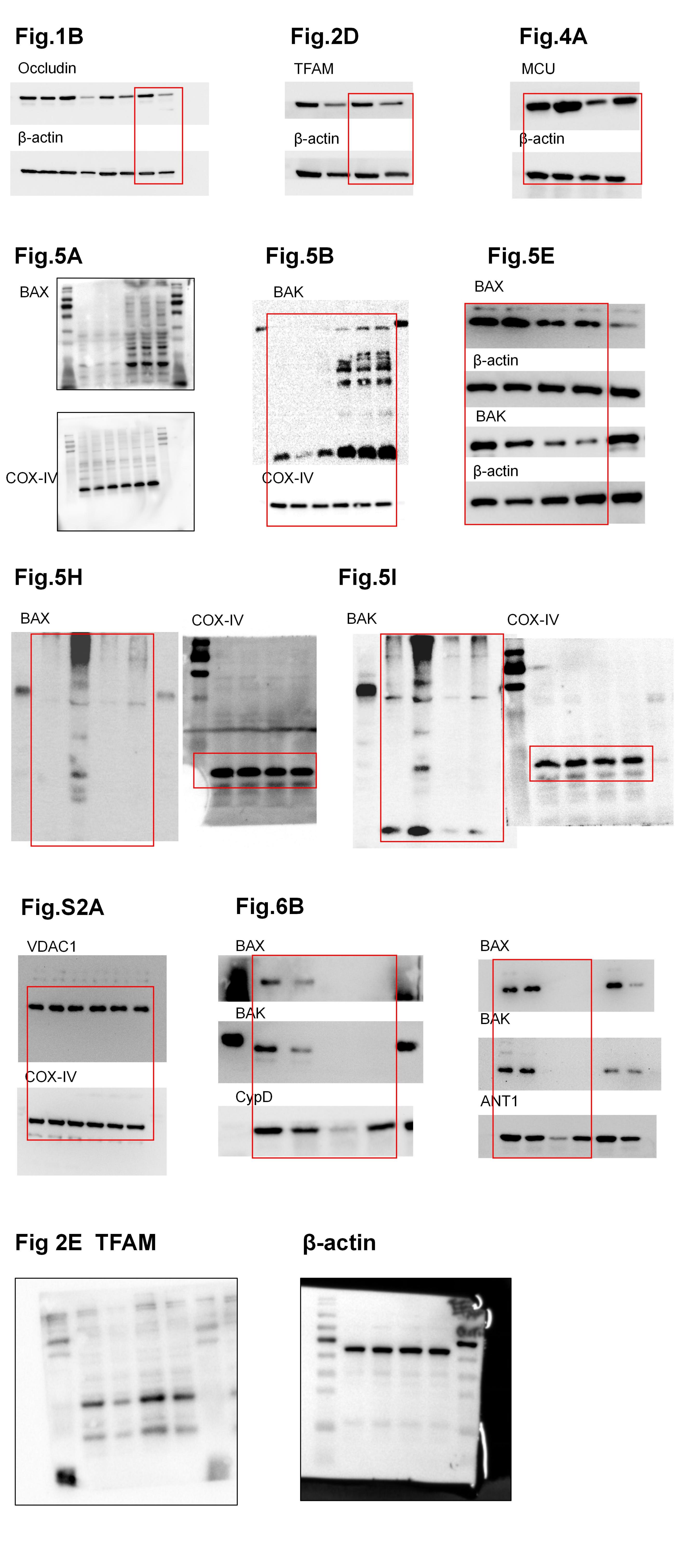

Supplement: Supplementary file 1 — Supplementary Material 1 [file 12964_2025_2603_MOESM1_ESM.jpg]
