# Supplementary material for: Mitochondrial DNA release via mPTP and BAX/BAK drives inflammatory injury in intestinal ischemia reperfusion
Source: Cell Commun Signal. 2025 Dec 24;24:50. doi: 10.1186/s12964-025-02603-3 (PMC12849455; doi:10.1186/s12964-025-02603-3)
Supplement: Supplementary file 2 — Supplementary Material 2 [file 12964_2025_2603_MOESM2_ESM.pdf]

figure.S1

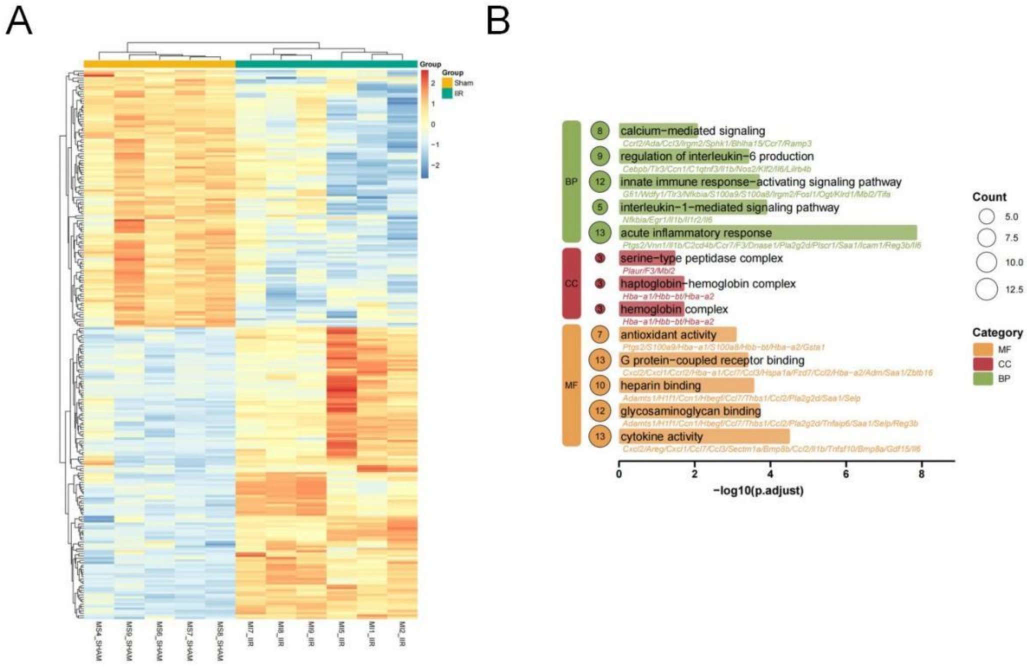

(A) Heatmap depicting differentially expressed IIR-related genes. The color represents the change in gene expression levels, with red indicating up-regulation and blue indicating down-regulation. The shade of the color reflects the degree of expression change. (B) GO enrichment analysis of differentially expressed genes was performed. Thevalue represents the significance of enrichment, with higher values indicating greater significance. The size of the circles represents the number of genes in each category.

figure.S2

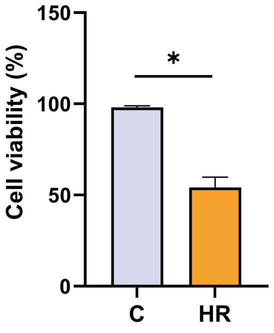

(A) Detection of cell viability in C and HR groups by CCK-8 assay, n = 6. Data are presented as mean ± SD.*P < 0.05.

figure. S3

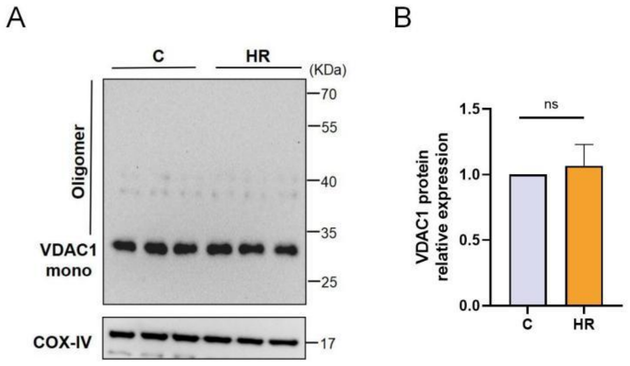

(A, B) Mitochondrial fractions were extracted through subcellular organelle separation. Western blot analysis of the expression levels of monomeric and polymeric forms of VDAC1 proteins in mitochondria after DSS cross-linking treatment, n = 6. Data are presented as mean ± SD. *P < 0.05.

figure. S4

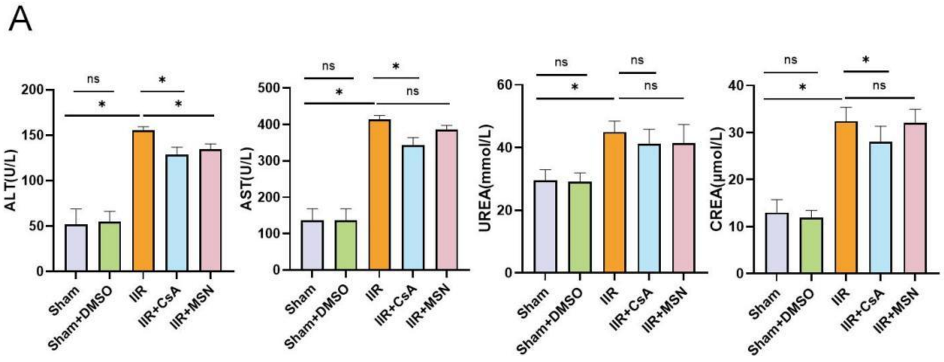

(A) Detection of the levels of ALT, AST, CREA, and UREA in murine plasma, n = 6. Data are presented as mean ± SD. *P < 0.05.
